# Narrative medicine as a teaching strategy for nursing students to developing professionalism, empathy and humanistic caring ability: a randomized controlled trial

**DOI:** 10.1186/s12909-023-04026-5

**Published:** 2023-01-18

**Authors:** Mengxin Xue, Huiping Sun, Jin Xue, Jingxin Zhou, Junchao Qu, Siqi Ji, Yuan Bu, Yongbing Liu

**Affiliations:** 1 School of Nursing and Public Health, Yangzhou University, Yangzhou, Jiangsu Province China; 2 Guangling College, Yangzhou University, Yangzhou, Jiangsu Province China

**Keywords:** Narrative medicine, Professionalism, Empathy, Humanistic care, Nursing students, Controlled trial

## Abstract

**Background:**

Narrative medicine has become a solution to cultivate medical students’ ability of empathy and humanistic care. However, the role of narrative medicine is lacking in the study of professionalism. The aim of this study was to analyze the effects of narrative medical theory learning and narrative writing on professionalism, empathy and humanistic care ability of nursing students.

**Methods:**

This cluster randomized controlled trial was conducted between June 2021 and June 2022 in two universities in Jiangsu, China. The participants of this study were 85 nursing students who were randomly divided into the intervention group (*n* = 43) or the control group (*n* = 42). Participants in the intervention group were trained in narrative medical theory learning and narrative writing based on a Web-based platform, while those in the control group were not. Self-report questionnaires of professionalism, empathy and humanistic care ability were used before and after intervention.

**Results:**

The results showed that the professionalism score of the intervention group was (68.7 ± 6.8 vs. 64.5 ± 7.5; *P* = 0.005), empathy (99.4 ± 15.7 vs. 92.2 ± 14.6; *P* = 0.014) and humanistic care ability (127.6 ± 20.0 vs. 113.3 ± 18.8; *P* = 0.004) were better than the control group.

**Conclusion:**

The results of this quantitative study suggest that narrative medical theory education and narrative writing based on the network platform can promote the development of professionalism, empathy and humanistic care ability of nursing undergraduates.

## Background

This study was guided by the theory of narrative medicine, first proposed by Rita Charon in 2001 and defined as the ability to acknowledge, absorb, interpret, and act on the stories and plights of others [1]. It is based on a model of empathy, reflection, professionalism and trust, and is inseparable from the concepts of empathy, humanism and professionalism [1]. Narrative medicine currently plays an important role in interdisciplinary learning, including in medicine, education, and the humanities, inspiring self-reflection and empathy [2], and which fosters narrative competence through curriculum content such as storytelling, careful reading, reflective writing, sharing, and discussion. To help healthcare professionals truly understand patients from different perspectives and experience their inner feelings in future clinical practice, so as to improve the level of humanistic literacy [2]. An innovative model of narrative medicine education should include training in writing skills [3]. On the one hand, one can benefit from training in narrative medicine (e.g., empathy) [4]. On the other hand, narrative writing in the form of storytelling can develop writing skills for personal and professional development [3]. In addition, the narrative medicine program includes potential benefits such as friendly interaction between faculty and students, narrative medicine writing skills, and understanding of ethical challenges [4].

Narrative-based medical interventions have a positive impact on medical students; such interventions are designed to help students hone their listening and observational skills, can stimulate self-reflection and empathy, help students think about patients from a different perspective, and are meaningful tools for motivating medical students’ professional and personal development [2]. At the same time, narrative medical education has been applied to the teacher population to provide a unique space for reflection on their growth [5]. In China, the implementation of narrative medicine education is often used as a course to help medical students improve their medical narrative skills, empathy, and humanistic care [6, 7]. In addition, narrative medicine education is often taught in reliance on a university course, such as Nursing Psychology, English course, and Surgical Nursing [8–10]. Through the combination of narrative medicine and university courses, the goal is to achieve simultaneous improvement of knowledge and skills, medical humanities and clinical empathy. Few studies have been conducted to implement narrative medicine education for nursing students during clinical practice. However, the period of clinical practice is an important time to develop nursing students’ professionalism, empathy, and humanistic care [11–13]. Therefore, providing a rich knowledge of medical humanities at this stage, enhancing medical empathy and humanistic care education and training, developing attention, listening, absorbing, interpreting, and responding to stories, etc., is important for the growth and development of future students’ careers.

During nursing education, educators are increasingly concerned about teaching professionalism [14]. Professionalism refers to the value orientation and standards of practicing nurse which reflects work attitude and concepts of nursing [15]. However, in the clinical environment, nursing students are faced with the high turnover rate of nurses, negative doctor-patient relationship, frequent workplace violence and other situations [16, 17], leading to cognitive deviation of nursing profession [18], which is not conducive to the cultivation of their professionalism. It has been reported that professionalism level can affect nurses’ job satisfaction and retention intention [19, 20]. In addition, De Santa et al. pointed out that clinical practice is the best way to cultivate and educate nursing students’ professionalism [11]. Therefore, developing the level of professional spirit during clinical practice has a positive impact on nursing students to continue to engage in this profession and alleviate the shortage of nurses.

Empathy is a multidimensional concept that includes emotional, cognitive, moral, behavioral and relational dimensions [21]. In the context of patient care, empathy has been defined as a cognitive attribute involving an understanding of patients’ experiences, concerns, and perspectives together with the ability to communicate this understanding and the intention to help [22], and is an important part of medical humanities education. Currently, there is a controversy about whether empathy decreases during the education of medical students. Hojat et al. showed that the level of empathy did not change during the first 2 years of medical education, however, at the end of the third year, medical students showed a significant decline in empathy and this decline persisted until graduation, notably, the weakening of empathy occurred when the course was directed towards patient activities [23]. In a longitudinal study, Julia Ward et al. found the greatest decline in empathy levels among those students with the most clinical exposure and those with healthcare work experience [12]. However, Roff et al. found that medical students’ empathy did not decline over time [24]. There is growing evidence that improved empathy during clinical practice increases patient adherence to treatment and patient satisfaction and builds positive relationships between nurses and patients, leading to more effective treatment outcomes [25, 26]. On the other hand, high levels of empathy can reduce stress and burnout [27, 28] and improve the quality of professional life [29]. Research has shown that empathy can be taught and trained through education and reflection [30]. Therefore, enhancing the level of empathy in nursing students is an important part of nursing education.

Jean Watson believed that nursing humanistic care ability was the nurses’ externalization of humanistic literacy into the clinical work and abilities to serve the patients consciously and creatively, and outlined 10 carative factors/caritas processes that include the development of humanistic-altruistic system of values, the development of helping-trusting human-caring relationship, the instillation and the enabling faith and hope, and the provision for a supportive, protective, and/or corrective mental, social, spiritual environment [31]. Nurses with high humanistic care ability can help reduce patients’ suffering related to disease, establish a good relationship with patients and their families, and better understand the meaning of their work [13]. Therefore, it is extremely important to cultivate nursing students to provide humanistic care behavior and caring ability for patients, and it is also the important responsibility and moral mission of teachers [32, 33].

As far as we know, there is a relative consensus on the efficacy of narrative medicine in cultivating empathy and humanistic care ability of medical students in both qualitative and quantitative studies, but the role of narrative medicine in professionalism in quantitative studies is lacking in the existing literature, which may be of significance to clinical practice and nursing education. In addition, our research focuses on bridging the gap in medical humanities education for undergraduate nursing students during their clinical practice period. We encourage students to combine theory and practice. With a strong theoretical foundation in narrative medicine, students can get a real sense of professionalism, empathy and humanistic care during their clinical placements with patients and healthcare professionals, and improve their own narrative writing skills. Therefore, this study designed a 12-month narrative medicine theory learning and narrative writing project to explore the application effect of narrative medicine in clinical practice on the professionalism, empathy ability and humanistic care ability of nursing students, as follows:

1. Does the narrative writing approach based on narrative medicine theory improve nursing students’ level of professionalism, empathy, and humanistic care ability in clinical practice?
2. Are there differences in the level of development of professionalism, empathy, and humanistic care ability of nursing students in the clinical setting with narrative writing methods based on narrative medicine theory compared to traditional practice logs?

## Methods

### Study design and methods of rigor

This study was in accordance with CONSORT guidelines and was a cluster randomized controlled trial. This study was conducted from June 2021 to June 2022 at two institutions of higher learning in Jiangsu, China. Cluster randomized controlled trial is a trial design in which study subjects are randomly assigned in clusters, which is designed to prevent sample contamination and improve participant compliance [34]. Before the nursing students entered clinical practice, the two schools held a meeting respectively, in which the same researcher explained the relevant information of the study to the nursing undergraduates in the form of face to face, and recruited nursing undergraduates who volunteered to participate in the study. At the end of the session, the participants all signed a written informed consent form. The cluster was defined as a school in this study and the random allocation of the intervention and control groups was carried out using a simple random grouping method (i.e. lottery). Two clusters are placed in two opaque envelopes and one envelope is randomly selected by one person, then that cluster is the intervention group and the other cluster is the control group.

### Participants

G*Power 3.1 was used to calculate the minimum sample size for analyzing differences between the two groups by a two-tailed test. With a statistical significance level of 0.05, power of 0.80, and effect size of 0.65, the sample size was calculated to be 39 for each group, totaling 78. Participants were fourth-year nursing undergraduates entering clinical practice. Nursing students who were able to complete the one-year internship required by the university and nursing students who did not take courses related to narrative medicine were eligible to participate in this study. Considering potential dropouts, we ultimately recruited 85 nursing students from both institutions (intervention group = 43, control group = 42), and all participants provided written and verbal informed consent.

### Procedure

**Fig. 1:**
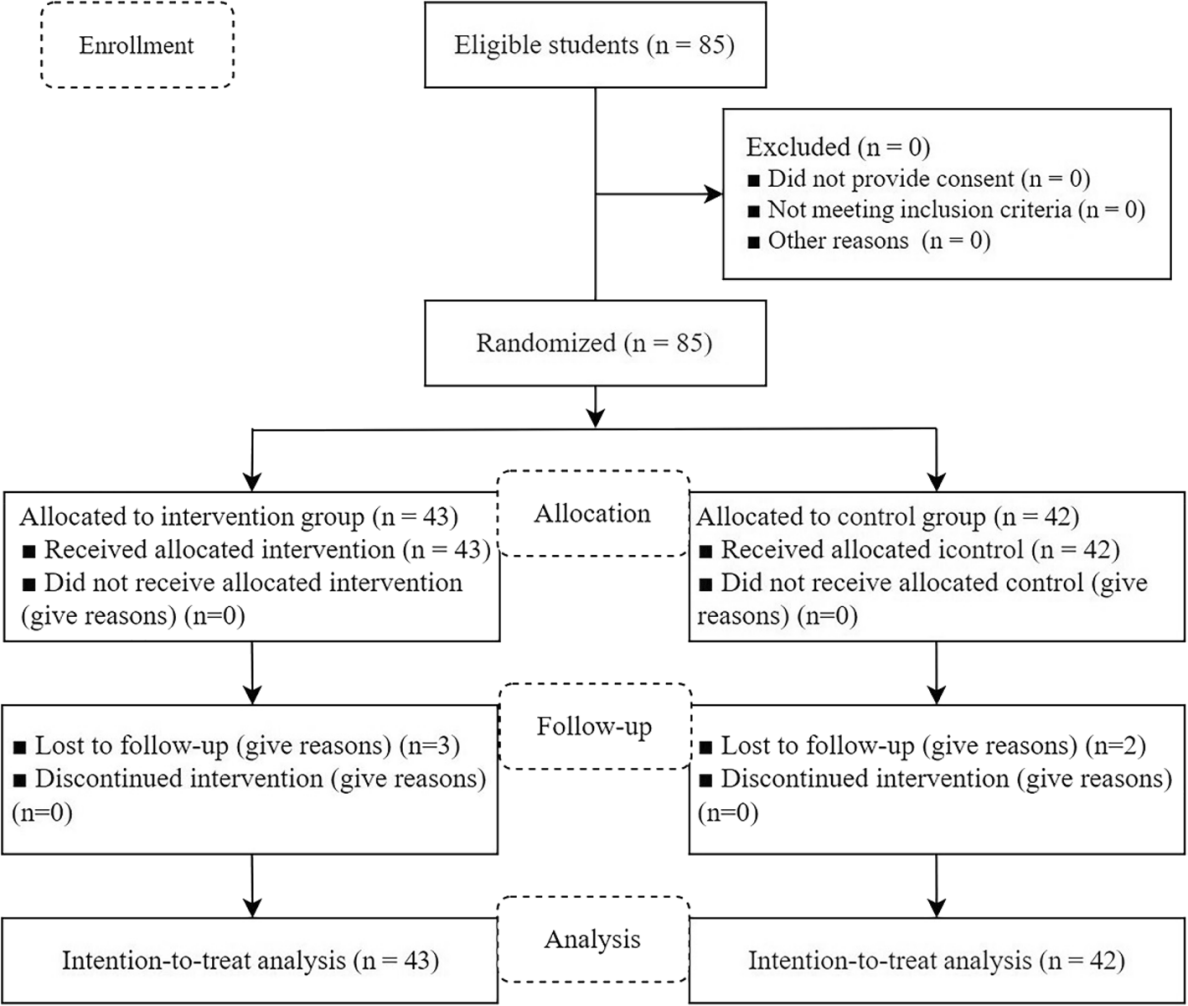
Flowchart of the inclusion of study participants according to CONSORT diagram

Researchers used demographic questionnaires, the professionalism scale, the empathy scale, and the humanistic care ability scale to examine baseline levels in both groups. Students in both the intervention and control groups were required by both schools to keep a minimum of one internship journal for 2 weeks. On this basis, the intervention group substituted the writing of internship logs through narrative medical theory courses and narrative writing, while the control group did not. At the end of clinical practice, the test results of the professionalism scale, empathy scale, and humanistic care ability scale were first compared between the two groups before and after the intervention, and secondly, the test scores were compared within the group before and after the intervention to assess the effectiveness of the intervention. We approached and assessed 85 fourth-year nursing students to participate in the study, with three students in the experimental group and two in the control group unable to complete the final assessment, as shown in Fig. 1.

### Narrative medicine program

Narrative medicine was introduced to the undergraduate nursing residency program, which focuses on narrative medicine seminars and narrative writing. First, participants attend a 3.5-hour narrative medicine seminar that focuses on giving students a preliminary understanding of narrative medicine and narrative writing. Then, we encourage nursing students to use narrative writing methods based on narrative medicine theory to record the stories between interns and patients, interns and medical staff, and medical staff and patients during clinical practice, and evaluate and share their narrative works. The purpose of this process is to cultivate the professionalism, empathy and humanistic care ability of nursing students. The narrative medicine model consists of a series of activities, described as follows:

#### Activity 1 Narrative medicine seminar

During clinical practice, we conduct a narrative medicine seminar. The conference consists of three parts, with students first receiving a 2-hour lecture on the theoretical background and empathy of narrative medicine. Then, students will read the narrative works carefully for 45 minutes to help them have a deep understanding of the narrative works. Finally, the students received a 45 min course and introduced the narrative writing content, as shown in Table 1.

**Table 1:** Course content of narrative medicine seminar

Theme	Goal	Content	Duration
Narrative medicine theory	Help students understand narrative medicine theory and lay the foundation for future narrative writing	(1) Definition and origin of narrative medicine. (2) Three focal points of narrative medicine. (3) Three elements of narrative medicine. (4) Two tools of narrative medicine.	1 h
Clinical empathy	Help students empathize with the patient’s story through narrative	(1) Empathic ability. (2) Performance of empathic behavior. (3) How to improve empathy by means of narrative.	1 h
Close reading of narrative works	Develop students’ narrative reading, observation, and listening skills.	(1) Reading of classic short stories and middle-length novels in narrative medicine. (2) Narrative work reading and empathy construction.	45 min
Narrative writing (reflective writing and parallel medical records)	Develop students’ narrative writing skills and enhance their reflective skills.	(1) Watching videos adapted from real cases. (2) Reflective writing/parallel medical records. (3) Writing Content.	45 min

#### Activity 2 Narrative writing theme

First, a researcher trained in a systematic course in narrative medicine talks to students about writing criteria (e.g., descriptions of time, place, people, reflections, etc.) so that nursing students know what narrative writing entails. Second, the evaluator explains the writing topic to the students, as shown in Table 2. During the writing process, nursing students chose their own topics and detailed their observations and reflections on their clinical experiences in narrative writing. One narrative writing was completed every 2 weeks, and students submitted the content and related pictures to an online web platform to facilitate timely feedback from the evaluators.

**Table 2:** Themes of narrative writing

Theme	Goal	Item
Patient and self	Help nursing students tell patients’ stories and understand patients’ feelings.	Please write down a patient experience that has impressed you
		Please write down a warm moment of interaction with your patient
		Please write down your understanding of the background of the patient’s illness
		Please write down the moment of your emotional disclosure
Medical staff and patient	Help nursing students to feel the relationship between medical staff and patients as an observer	Please write down your most rewarding moment
		Please write down what you think is a good doctor-patient relationship
		Please write down the humanistic behaviors you see in the medical staff
Medical staff and self	To help nursing students enhance their professional understanding through contact with medical staff	Write down the qualities or behaviors of the people you admire most in the clinical staff
		Please write about your caring moments with the medical staff
		Please write about what you know about this medical career or what actions have influenced your view of the profession with a new perspective.

#### Activity 3 Evaluation of narrative works

After the nursing students submit their narrative writing, researchers will evaluate and give feedback on their narrative writing every 2 weeks through the online platform according to the writing standards, so that the nursing students can receive feedback and correct their writing in time. For example, the narrative writing submitted by the nursing students did not adequately describe their inner feelings and did not reflect on their own behaviors. Researchers should guide the nursing students to think by asking questions, such as, “What was your first feeling when you knew the experience of the patient?”. Unqualified narrative writing should be returned for revision, such as misinterpreting narrative writing as simply describing the events of the day or week. This activity aims to improve the quality of nursing interns’ narrative writing, ensuring that students have descriptions, feelings and reflections in narrative writing.

#### Activity 4 Sharing of narrative works

The research team establishes a column of excellent narrative works, and two researchers select 2–5 narrative works and send them to the column every 2 weeks. If there are some incomplete or sentence problems in the narrative works, researchers need to submit suggestions for modification to the nursing students, and the works will be pushed to the column after modification and improvement. Finally, researchers shared this column with nursing students. The purpose of this sharing activity is to promote nursing interns to better understand the patient experience and the reflective experience of other students, and to play a role of mutual learning.

### Measures

#### The professionalism scale

Nursing professionalism was assessed using Chinese version of Hall’s Professionalism Inventory. The inventory was originally designed to determine suitable criteria and measure a person’s professional standing by Professor Richard Hall in 1968 [35]. Wu translated and revised the scale into Chinese in 2019, which was proved to have good reliability and validity, and was applied to Nursing students in China [36]. The Chinese inventory consisted of six subscales including professional organization, public service, self-discipline, autonomy, sense of mission and job satisfaction and a total of 20 items. Every item is rated on a 5-point Likert-scale (1 = totally disagree to 5 = totally agree). Higher scores indicates higher level of nursing professionalism. The Cronbach’s α coefficient was 0.750 and the test-retest reliability was 0.840 [36]. In this study, Cronbach’s alpha was 0.729.

### The Jefferson scale of empathy for nursing students (JSPE-NS)

We used a nursing student version of the Jefferson Scale of Empathy to assess nursing students’ empathy. The scale, developed by Hojat et al. [37], is divided into three dimensions of perspective taking, compassionate care, and standing in patient’s shoes, with 20 items. Every item is rated on a 7-point Likert-scale (1 = totally disagree to 7 = totally agree). Scores range from 20 to 140. The Cronbach’s α coefficient and construct validity of the Chinese version of JSPE-NS are 0.74 and 0.89, respectively [38]. In this study, Cronbach’s alpha was 0.868.

### The humanistic caring ability of nursing undergraduates

The evaluation of students’ humanistic care ability adopts the humanistic care ability scale of nursing students compiled by Huang Gebing. The scale is based on Watson’s humanistic care theory and its 10 caring factors [39], which contains eight dimensions: Instilling faith and hope, health education, humane and altruistic values, scientifically solving health problems, helping to meet basic needs, providing a good environment, promoting emotional communication, helping to solve difficulties, a total of 45 items. The scale adopted likert 5-level score (0 = completely inconsistent to 4 = completely consistent), with scores ranging from 0 to 180. The higher the score, the better the humanistic care ability. The Cronbach’s α coefficient and content validity of the scale were 0.904 and 0.960, with good reliability and validity [40]. It has been widely used in nursing students in China. In this study, Cronbach’s alpha was 0.952.

### Statistical analysis

The data were analyzed using IBM SPSS Version 26. Statistical analysis also uses intention-to-treat analysis (ITT) to process missing data [41]. The categorical variables were described using frequency and percentage, and the continuous variables were described using mean (standard deviation). Pearson chi-square test was used to analyze the differences of categorical variables. If the variables were normally distributed, the difference between the two groups of continuous variables was tested using the independent sample T test; otherwise, the Mann-Whitney U nonparametric test was used. Paired T test or Wilcoxon rank sum test were used to analyze the differences between the two groups before and after intervention.

## Results

### Comparison of baseline characteristics

The mean age of the 85 participants was 21.5 ± 1.2 years, of which 13 (15.3%) were male and 72 (84.7%) were female. There were no significant differences between the intervention group and the control group in age, gender, family background, only child, student cadre and internship team leader (*P* > 0.05). In addition, there were no significant differences between the two groups in the baseline scores of professionalism, empathy, and humanistic care ability before the intervention (*P* > 0.05), as shown in Table 3.

**Table 3:** Comparison of baseline characteristics between the control group and the intervention group

Variables	Control group (*n* = 42)	Intervention group (*n* = 43)	χ^2^/Z	*P* value ^a^
Age, mean (SD)	21.4 (0.9)	21.5 (1.4)	−0.724	0.469
Gender, N (%)				
Males	7 (16.7)	6 (14.0)	0.121	0.728
Females	35 (83.3)	37 (86.0)		
Family background, N (%)				
Urban	21 (50.0)	20 (46.5)	0.104	0.784
Rural	21 (50.0)	23 (53.5)		
Only child, N (%)				
Yes	21 (50.0)	25 (58.1)	0.567	0.451
No	21 (50.0)	18 (41.9)		
Student cadre, N (%)				
Yes	19 (45.2)	28 (65.1)	3.396	0.065
No	23 (54.8)	15 (34.9)		
Internship team leader, N (%)				
Yes	7 (16.7)	9 (20.9)	0.253	0.615
No	35 (83.3)	34 (79.1)		
Professionalism, mean (SD)	63.8 (6.2)	65.0 (7.8)	−0.337	0.736
Empathy, mean (SD)	88.7 (11.9)	89.6 (14.0)	−0.373	0.709
Humanistic care ability, mean (SD)	114.3 (20.6)	117.5 (22.3)	−0.503	0.615

^a^ Mann-Whitney U test results for continuous variables with abnormal distribution, and Chi-square test results for categorical variables

### Comparison between the two groups after intervention

**Fig. 2:**
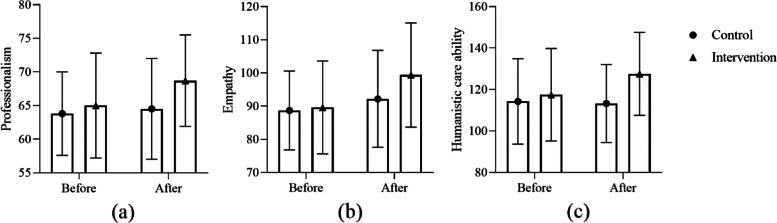
The scores of professionalism, empathy and humanistic care of nursing students before and after intervention were compared between the two groups

Compared with the nursing students in the control group, the professionalism (68.7 ± 6.8 vs. 64.5 ± 7.5; *P* = 0.005), empathy (99.4 ± 15.7 vs. 92.2 ± 14.6; *P* = 0.014) and humanistic care ability (127.6 ± 20.0 vs. 113.3 ± 18.8; *P* = 0.004) of the students in the intervention group were significantly improved, as shown in Table 4. Figure 2 shows the comparison of professionalism, empathy and humanistic care ability scores between the two groups before and after the intervention.

**Table 4:** Outcomes of 85 nursing students between intervention and control group, mean (SD)

Variables	Control group (*n* = 42)	Intervention group (*n* = 43)	Z	*P* value ^a^
Professionalism	64.5 (7.5)	68.7 (6.8)	−2.816	0.005
Empathy	92.2 (14.6)	99.4 (15.7)	−2.455	0.014
Humanistic care ability	113.3 (18.8)	127.6 (20.0)	− 2.844	0.004

^a^ Mann-Whitney U test

### Intra-group comparison before and after intervention

We also analyzed the comparison of pre - and post-intervention scores between the intervention and control groups. Data showed that from baseline to intervention, scores of professionalism (63.8 ± 6.2 vs. 64.5 ± 7.5; *P* = 0.431), empathy (88.7 ± 11.9 vs. 92.2 ± 14.6; *P* = 0.072) and humanistic care ability (114.3 ± 20.6 vs. 113.3 ± 18.8; *P* = 0.886) in the control group showed no significant differences, while scores of professionalism (65.0 ± 7.8 vs. 68.7 ± 6.8; *P* = 0.003), empathy (89.6 ± 14.0 vs. 99.4 ± 15.7; *P* = 0.001) and humanistic care ability (117.5 ± 22.3 vs. 127.6 ± 20.0; *P* = 0.025) in the intervention group were significantly improved, as shown in Table 5.

**Table 5:** Baseline and follow-up scores for the intervention and control groups, mean (SD)

Group	Variables	Pre-test	Post-test	t/Z	*P* value
Control	Professionalism	63.8 (6.2)	64.5 (7.5)	−0.795	0.431^a^
	Empathy	88.7 (11.9)	92.2 (14.6)	−1.8	0.072^b^
	Humanistic care ability	114.3 (20.6)	113.3 (18.8)	−0.143	0.886^b^
Intervention	Professionalism	65.0 (7.8)	68.7 (6.8)	−3.135	0.003^a^
	Empathy	89.6 (14.0)	99.4 (15.7)	−3.236	0.001^b^
	Humanistic care ability	117.5 (22.3)	127.6 (20.0)	−2.326	0.025^a^

^a^ T test; ^b^ Wilcoxon test

## Discussion

The results of this study confirm that after 12 months of intervention, participants assigned to the narrative medicine program showed more favorable changes in self-reported levels of professionalism than the control group. In addition, pre-test and post-test results in the intervention group showed better results in terms of professionalism. Therefore, the results of this study confirm that narrative medical theory education and narrative writing play a good role in the training of professionalism in nursing students. Previous qualitative research has suggested that narrative medicine has been found to positively influence the development of professional identity and professional literacy [42, 43]. Narrative-based medical education promote medical interns’ perceptions of self-exploration, reflection, and awareness of professional identity, thereby influencing their learning of professionalism in clinical settings [42]. During the narrative writing process, participants in the intervention group observe or describe their interactions with patients and the behaviors of clinical healthcare professionals from a reflective perspective, thus sharing the new knowledge and skills they have learned, as well as their perceptions and outlook on values, sense of mission, and professional identity [42, 44]. Participants in the control group, on the other hand, would not, and they often stood to describe changes in their own professional skills more from their own perspective. In addition, the role of a role model or mentor influences the development of students’ professionalism [45]. We set up writing themes to inspire students to look closely at the role model behaviors of health care professionals or mentors in clinical practice and write about them through narrative medicine. The content of these writings provokes students to reflect on their career paths, such as their responsible attitude towards patients and their work, and their passion for nursing. Therefore, reflective practice through writing is necessary for the development of students’ professionalism.

In our study, after the intervention, the empathic ability of the intervention group was better than that of the control group. Also, the pre-test and post-test results of the intervention group showed a significant improvement in empathic ability. Therefore, students who received the narrative medicine education program showed more empathy than students who did not receive the program, and the results were consistent with previous research [4, 46]. On the one hand, narrative theory educational learning can promote student empathy [4]. This may promote positive empathy among clinical staff by exploring literature [47]. Previous studies have shown [9, 48] that medical professionals’ participation in narrative medicine programs leads to increased self-reported empathy scores. The three elements of narrative medicine are attention, reproduction, and belonging. By focusing on the patient, writing the patient’s story, and in the process being able to feel what the patient feels and understand the patient’s perspective; attention and reproduction spiral into a belonging relationship between the doctor and the patient, nursing interns are able to examine their own behavior and thoughts in this process, understand the patient, and empathize with them [9]. On the other hand, writing may be an important way to enhance empathy and by recording the patient’s story, students have more perspective to contact the patient’s emotions and gain a deeper understanding and appreciation of the patient as a way to achieve empathy [49]. However, one study reported only a small increase in participants’ empathy levels 1 year after the narrative medicine workshop [2]. Therefore, future research on how to maintain a steady increase in empathy levels through continuous training is essential.

The results of the study showed that after 12 months of narrative medicine education, the humanistic care competencies of the intervention group were significantly higher than those of the control group. At the same time, the pre-test and post-test results of the intervention group were significantly higher in humanistic care competence, which is consistent with previous studies [50]. Therefore, narrative medical theory education and narrative writing models help to improve students’ humanistic care, which may be due to a variety of reasons. Jeffrey Lorenz pointed out that the cultivation of humanism has two key elements of medicine, one by building relationships with patients and the other by setting aside time for reflection through narrative creation, however, this is not present in most clinical rotations [51]. In our study, we aim to encourage students to record their stories with patients and healthcare professionals and to create narratives in which they can feel and reflect in order to improve their personal humanistic care. Rita Mustika et al. believe that role models play an important role in the cultivation of humanism [52]. Students actively observe the humanistic behaviors of clinical medical staff, actively explore the effects and values of these behaviors through description and reflection, and eventually become their new behaviors. This helps to develop students’ humanistic values and behaviors. In addition, studies have shown that nursing students’ perception of organizational caring atmosphere can directly affect their humanistic care ability [53]. It is not only the caring behavior of clinical medical staff for patients and their families, but also the caring behavior of medical staff for interns and colleagues. By setting the theme of writing, we guide nursing students to pay attention to caring behaviors around them, which may make it easier for them to practice humanistic behaviors, so as to realize humanistic care for patients.

Therefore, this study used a combination of medical narrative theory training and narrative writing to develop students’ narrative writing and reflection skills in order to enhance professionalism, empathy, and humanistic care among undergraduate nursing students. After a 12-month intervention study, the importance of narrative medicine in medical education in developing students’ professionalism, empathy and humanistic care, especially in clinical practice, was confirmed.

### Study limitations

This study is based on the writing requirements of the original internship diary into a narrative writing method based on narrative medicine theory. As far as we know, most schools in China require interns to submit internship logs, which may be beneficial to our narrative medicine intervention project. But the study also has the following limitations. First, our study used voluntary participation and self-report survey methods, which may be biased. Second, our study only investigated nursing undergraduates who participated in clinical practice, so we can further study nursing students of all grades in the future. Third, all nursing students are required to complete a two-week internship diary or narrative writing based on narrative medical theory as part of their fourth-year internship assignment, which may lead to bias. Fourth, this study lacks an assessment of the long-term impact of narrative medicine programs, which may have implications for the development of a longitudinal intervention program to maintain a steady increase in student professionalism, empathy, and humanistic care ability. Therefore, there is a need to evaluate the long-term outcomes of narrative medical education and narrative writing programs in future studies.

## Conclusion and practice implications

The results of this quantitative study demonstrate the benefits and value of a web-based platform for narrative medical theory education and narrative writing to promote the development of professionalism, empathy, and humanistic care ability in nursing students. This study used narrative medical theory training and writing themes to guide students in reflecting on a number of clinical performances and behaviors that lead to higher quality care for patients. Thus, narrative medicine, as an interdisciplinary field, can improve the humanistic component of medical education that is in short supply, which has the potential to benefit personal and professional development. Furthermore, in order to achieve the desired results, we must prioritize the enabling conditions that will benefit the development of narrative medicine projects. Narrative medical education is a novel teaching method that can be used to improve students’ narrative writing and reflective skills through writing, and is a low-cost, easily shared learning tool. As far as we know, most institutions in China have a requirement to keep an internship journal, and these circumstances are more conducive to the development of narrative medical education. The intervention program in this study is easy to implement and replicable. These promote educational reform in clinical and nursing disciplines, bridge the gap in medical humanities education for nursing students during clinical practice, and provide some reference for the development of medical humanities literacy.

## Data Availability

The datasets used and analyzed during the current study can be provided by the corresponding author on a reasonable request.

## References

[1] Charon R (2001). The patient-physician relationship. Narrative medicine: a model for empathy, reflection, profession, and trust. Jama..

[2] Milota MM, van Thiel G, van Delden JJM (2019). Narrative medicine as a medical education tool: a systematic review. Med Teach.

[3] Remein CD, Childs E, Pasco JC (2020). Content and outcomes of narrative medicine programmes: a systematic review of the literature through 2019. BMJ Open.

[4] Daryazadeh S, Adibi P, Yamani N (2020). Impact of narrative medicine program on improving reflective capacity and empathy of medical students in Iran. J Educ Eval Health Prof.

[5] Holdren S, Iwai Y, Lenze NR, et al. A novel narrative medicine approach to DEI training for medical school faculty. Teach Learn Med. 2022:1–10. 10.1080/10401334.2022.2067165 35608161

[6] Yang NX, Li XY, Yan H (2018). Influence of narrative medical education on empathy ability and academic achievement of clinical medical students: a randomized controlled trial. Chinese J Clin Psychol.

[7] Shi HR, Zhou X, Shan BF (2021). The practice and effects of narrative nursing course for nursing undergraduates. Chinese J Nurs Educ.

[8] Wang YB, Wu SL, Wang L (2021). Discussion on the application of narrative medicine in nursing psychology teaching. Med Philos.

[9] Li W, Li CJ, Chen BB (2022). Exploration and practice of narrative medicine education on promoting medical Students’Empathy ability. Chinese Health Ser Manage.

[10] Shan YW, Wu KL, Sun YN (2021). Application of moral education online teaching of surgical nursing science based on the medical narrative concept in undergraduate nursing teaching. Chin Nurs Res.

[11] De Santa MP, Podder BL (2016). Nursing student’s clinical learning experiences and the barriers faced. Int J Nurs Educ.

[12] Ward J, Cody J, Schaal M (2012). The empathy enigma: an empirical study of decline in empathy among undergraduate nursing students. J Prof Nurs.

[13] Wu HL, Volker DL (2012). Humanistic nursing theory: application to hospice and palliative care. J Adv Nurs.

[14] Baernstein A, Fryer-Edwards K (2003). Promoting reflection on professionalism: a comparison trial of educational interventions for medical students. Acad Med.

[15] Duphily NH (2014). Simulation education: a primer for professionalism. Teach Learn Nurs.

[16] Liu W, Zhao S, Shi L (2018). Workplace violence, job satisfaction, burnout, perceived organisational support and their effects on turnover intention among Chinese nurses in tertiary hospitals: a cross-sectional study. BMJ Open.

[17] Wang M, Liu GG, Zhao H (2020). The role of mediation in solving medical disputes in China. BMC Health Serv Res.

[18] LZ, MX (2016). Current situation investigation and countermeasures of medical students' professional identity. Xuexiao Dangjian Yu Sixiang Jiaoyu.

[19] Hwang JI, Lou F, Han SS (2009). Professionalism: the major factor influencing job satisfaction among Korean and Chinese nurses. Int Nurs Rev.

[20] Shin SY, Kim JH (2021). Factors influencing retention intention of nurses at long-term care hospitals in Korea. J Gerontol Nurs.

[21] Derksen F, Bensing J, Lagro-Janssen A (2013). Effectiveness of empathy in general practice: a systematic review. Br J Gen Pract.

[22] Hojat M, DeSantis J, Gonnella JS (2017). Patient perceptions of Clinician's empathy: measurement and psychometrics. J Patient Exp.

[23] Hojat M, Vergare MJ, Maxwell K (2009). The devil is in the third year: a longitudinal study of erosion of empathy in medical school. Acad Med.

[24] Roff S (2015). Reconsidering the "decline" of medical student empathy as reported in studies using the Jefferson scale of physician empathy-student version (JSPE-S). Med Teach.

[25] Kim SS, Kaplowitz S, Johnston MV (2004). The effects of physician empathy on patient satisfaction and compliance. Eval Health Prof.

[26] Moreno-Poyato AR, Rodríguez-Nogueira Ó (2021). The association between empathy and the nurse-patient therapeutic relationship in mental health units: a cross-sectional study. J Psychiatr Ment Health Nurs.

[27] Yao X, Shao J, Wang L (2021). Does workplace violence, empathy, and communication influence occupational stress among mental health nurses?. Int J Ment Health Nurs.

[28] Cheng L, Yang J, Li M (2020). Mediating effect of coping style between empathy and burnout among Chinese nurses working in medical and surgical wards. Nurs Open.

[29] Hui Z, Dai X, Wang X (2020). Mediating effects of empathy on the association between nursing professional values and professional quality of life in Chinese female nurses: a cross-sectional survey. Nurs Open.

[30] Paloniemi E, Mikkola I, Vatjus R (2021). Measures of empathy and the capacity for self-reflection in dental and medical students. BMC Med Educ.

[31] Watson J. Watson's theory of human caring and subjective living experiences: Carative factors/caritas processes as a disciplinary guide to the professional nursing practice. Texto & Contexto Enfermagem. 2007;16(1):129–35.

[32] Lee-Hsieh J, Kuo CL, Tseng HF (2005). Application and evaluation of a caring code in clinical nursing education. J Nurs Educ.

[33] Beck CT (1992). Caring among nursing students. Nurse Educ.

[34] Campbell MK, Piaggio G, Elbourne DR (2012). Consort 2010 statement: extension to cluster randomised trials. Bmj..

[35] Hall. (1968). Professionalization and bureaucratization. Am Sociol Rev.

[36] Wu LF. Studies on the status of the attitude of professionalism, humanistic care ability and social support of nursing undergraduates and their relationships: Yangzhou University; 2019.

[37] Ward J, Schaal M, Sullivan J (2009). Reliability and validity of the Jefferson scale of empathy in undergraduate nursing students. J Nurs Meas.

[38] ZQ (2010). Study on the empathy status for higher vocational nursing students in Hunan Province.

[39] JW (1985). Nursing:Human science and human care: a theory of nursing.

[40] YH (2007). An empirical study on evaluation of humanistic Cadng ability of nursing undergraduates.

[41] Fisher LD, Dixon DO, Herson J (1989). Intention to treatment in clinical trials. Issues in pharmaceutical drug development.

[42] Huang CD, Jenq CC, Liao KC (2021). How does narrative medicine impact medical trainees' learning of professionalism? A qualitative study. BMC Med Educ.

[43] Miller E, Balmer D, Hermann N (2014). Sounding narrative medicine: studying students' professional identity development at Columbia University College of physicians and surgeons. Acad Med.

[44] Dhaliwal U, Singh S, Singh N (2018). Reflective student narratives: honing professionalism and empathy. Indian J Med Ethics.

[45] Jack K, Hamshire C, Chambers A (2017). The influence of role models in undergraduate nurse education. J Clin Nurs.

[46] Arntfield SL, Slesar K, Dickson J (2013). Narrative medicine as a means of training medical students toward residency competencies. Patient Educ Couns.

[47] Lanocha N. Lessons in stories: why narrative medicine has a role in pediatric palliative care training. Children (Basel). 2021;8(5):321. 10.3390/children8050321 PMC8143552 33922034

[48] Chen PJ, Huang CD, Yeh SJ (2017). Impact of a narrative medicine programme on healthcare providers' empathy scores over time. BMC Med Educ.

[49] Chen I, Forbes C (2014). Reflective writing and its impact on empathy in medical education: systematic review. J Educ Eval Health Prof.

[50] Hua QQ, Dai XY, Yi XH (2022). Cultivating path of medical Students' humanistic care ability from the perspective of narrative medicine. Med Philos.

[51] Lorenz FJ, Darok MC, Ho L, et al. The impact of an unconventional elective in narrative medicine and pediatric psycho-oncology on humanism in medical students. J Cancer Educ. 2022; 37(6):1798–805. 10.1007/s13187-021-02029-8 34057696

[52] Mustika R, Soemantri D (2020). Unveiling the hurdles in cultivating humanistic physicians in the clinical setting: an exploratory study. Malays J Med Sci.

[53] SL, ZD, ZL (2021). The mediating effect of emotional intelligence on perceived organizational care climate and humanistic care of undergraduate nursing students. J Nurs.

